# Collective effects of the cost of opinion change

**DOI:** 10.1038/s41598-020-70809-1

**Published:** 2020-08-14

**Authors:** Hendrik Schawe, Laura Hernández

**Affiliations:** Laboratoire de Physique Théorique et Modélisation, UMR-8089, CNRS-CY Cergy Paris Université, Cergy-Pontoise, France

**Keywords:** Complex networks, Phase transitions and critical phenomena

## Abstract

We study the dynamics of opinion formation in the situation where changing opinion involves a cost for the agents. To do so we couple the dynamics of a heterogeneous bounded confidence Hegselmann–Krause model with that of the resources that the agents invest on each opinion change. The outcomes of the dynamics are non-trivial and strongly depend on the different regions of the confidence parameter space. In particular, a second order phase transition, for which we determine the corresponding critical exponents, is found in the region where a re-entrant consensus phase is observed in the heterogeneous Hegselmann–Krause model. For regions where consensus always exist in the heterogeneous Hegselmann–Krause model, the introduction of cost does not lead to a phase transition but just to a continuous decrease of the size of the largest opinion cluster. Finally in the region where fragmentation is expected in the heterogeneous HK model, the introduction of a very small cost surprisingly increases the size of the largest opinion cluster.

## Introduction

Mathematical models of opinion dynamics aim in general to understand the systemic consequences of a dynamics based on interactions among agents derived from Social Influence Theory^1^. In this framework a large variety of models have been studied: discrete—binary or multiple—or continuous opinion variables, scalar or vector opinions, mixed or networked population^2,3^.

The influence of quenched disorder in the form of idiosyncratic properties of the agents has also been studied, like the inclusion of a proportion of stubborn or militant agents in the society^4–6^, or the role of the heterogeneity in the confidences that agents hold on each other^7–14^. Very recently we have presented a numerically extensive study on the role of heterogeneous quenched confidences on the bounded confidence Hegselmann–Krause (HK) model^15^, which will be the starting point of the work we present here.

In most cases, these works include in their models two basic psychological aspects, conceptualized by Deutsch and Gerard^16^, that lead the agents to conform to the opinion of others. This is often modelled by *social influence*, the tendency of an agent to adapt to the opinions of agents it interacts with, and *homophily*, the fact that an agent interacts more with those that are more alike. One of the great successes of the mathematical modelling of opinion formation in terms of a dynamical system was precisely to understand how we can observe different opinions coexisting in a society, despite the fact that the two considered interactions tend to homogenize the opinions of the social actors^17^.

This scheme is well adapted to understand the dynamics of opinion formation about many subjects of social life. However there are situations where modifying its own opinion may involve a particular effort for the agent, to the extent that if the agent cannot afford this effort, then it will be impossible for it to evolve. Social issues of this kind are those where the change of opinion automatically involves a behavioural change. This is not always the case: changing the opinion about the literary qualities of an author after a discussion with a well informed friend is one thing, but let us consider a subject like climate change. In this case, passing from a careless point of view to a more strict position concerning the preservation of the environment, does involve an effort to fundamentally change one’s behaviour. It can be said that in this kind of situation, changing one’s opinion bears a *cost*. This cost may be strictly economic, like spending more money on locally produced food instead of cheap food produced on an area which was, until recently, rain forest. Or it may be just behavioural, like using the train instead of travelling by plane, which incurs the cost of an increased travel time but is less harmful for the environment. The model we introduce in this article addresses situations where agents have a fixed initial amount of resources from which they have to pay each time they change their opinion. In this way the resources of each agent decrease as their opinions evolve.

One of the motivations for studying the role of the cost of opinion change is to try to understand one of the aspects of the theory that Anthony Downs postulated to describe the behaviour of public attention face to some issues that can dramatically affect social life, known as *Downs’ Attention cycle*^18^. When a society faces a problem that affects at the beginning a small fraction of its members but that carries a potential threat to the whole society, according to Downs, the public interest towards the problem will undergo five phases:

1. Pre-problem stage. Only a small fraction of the population is aware of the problem, either because they are affected or because they are specialists.
2. Alarmed discovery and euphoric enthusiasm. More and more social actors think that the problem should be addressed.
3. Realizing the cost of significant changes. Social actors realize that a big effort (either economic or behavioural) is required to solve the problem.
4. Gradual decline of intense public interest. Upon realizing of the high cost of necessary changes, most actors become discouraged and do not support the changes anymore.
5. The post-problem stage. Although social interest has been re-directed to other urgent problems, the global attention devoted to solve the original one is still higher than in the pre-problem stage.

In this work we concentrate on the notion of cost associated to the opinion change that intervenes in phases 3 and 4 of Downs’ attention cycle. We want to understand to what extent taking into consideration the cost of opinion change modifies the outcomes of the dynamics. Therefore we introduce a simple model that couples the dynamics of a heterogeneous bounded confidence HK model to that of the resources of the agents, which decrease as they are invested in sustaining successive opinion changes.

The article is organized as follows: in “Models and methods” we introduce the model and the methods applied, in “Results” we describe the results and discuss them in corresponding subsections, and finally the conclusions and perspectives are presented in “Conclusion”.

## Models and methods

In the heterogeneous Hegselmann–Krause model^19,20^ each agent *i* is described by a continuous dynamical variable, $x_i(t) \in [0, 1]$, representing the state of its opinion about a given subject and a quenched variable $\varepsilon _i$ that represents its *confidence*. The fundamental assumption of *bounded confidence* models is that one agent only interacts with those who already have a similar opinion. The confidence $\varepsilon _i$ then determines to what extent the opinion of another agent may differ from that of agent *i* for the interaction to take place. More formally the set of interaction partners, i.e. *neighbours*, of agent *i* is

1 $$\begin{aligned} I(i, \mathbf {x}) = \left\{ 1 \le j \le n | \left| x_i - x_j\right| \le \varepsilon _i \right\} . \end{aligned}$$

Note that this definition includes agent *i* as a neighbour of itself and in the case of heterogeneous confidences, i.e. $\varepsilon _i \ne \varepsilon _j$, the possibility of interaction between two agents is not symmetric.

The dynamics is defined in discrete time. At each time step a synchronous update of all agents is performed, in which every agent takes the average opinion of all its neighbours

2 $$\begin{aligned} x_i(t+1) = \frac{1}{N_i(\mathbf {x}(t))} \sum _{j\in I(i, \mathbf {x}(t))} x_j(t) \end{aligned}$$

where $N_i(\mathbf {x}(t))$ is the number of neighbours of agent *i*, the cardinality of $I(i, \mathbf {x})$.

This particular model leaves the initial conditions and the confidences as free parameters. In this manuscript we will always use random initial conditions of opinions, $x_i(0)$, drawn uniformly from [0, 1]. The most studied case considers that all the agents have the same level of confidence, i.e. $\varepsilon _i = \varepsilon \; \forall i$. In this case all agents converge to a single *consensus* opinion for $\varepsilon \gtrsim 0.2$^20^. Below this threshold the agents converge either to a *polarized* state of two dominant opinions or *fragment* into many opinion clusters.

In order to introduce heterogeneity in the confidences of our model society, we draw the $\varepsilon _i$ from a uniform distribution bounded by two parameters, $\varepsilon _l$ and $\varepsilon _u$, which allows us to tune how open or closed minded the society is: the lower bound, $\varepsilon _l$, fixes the lowest confidence value of the most closed minded agents and the upper bound, $\varepsilon _u$, the highest confidence value of the most open minded ones. Their difference can be interpreted as the degree of heterogeneity of confidences in the society. Note that the choice to use a uniform distribution of confidences between these values was made, since we have already studied this case in depth^15^, but studies for other choices, like a bimodal society where each agent has either a confidence value of $\varepsilon _l$ or $\varepsilon _u$, also exist^7,9^, as well as other distributions^15^, which all behave similarly.

We extend the heterogeneous HK model by integrating the *cost* that the agent has to afford (whatever the origin of such cost is) to update its opinion. Each agent is endowed with a limited amount of resources, $c_i(0)$, assigned to it at the beginning of the simulation. The opinion update of Eq. (2) takes place as long as the agent has resources to afford the change, otherwise we cap the change to the maximum which can be afforded by the agent:

3 $$\begin{aligned} x_i(t+1) = {\left\{ \begin{array}{ll} x_i' &{}\text { if }\,\, \eta \left| x_i(t) - x_i'\right| \le c_i(t)\\ x_i(t) + \frac{c_i(t)}{\eta } {{\,\mathrm{sgn}\,}}( x_i' - x_i(t) ) &{}\text { otherwise } \end{array}\right. } \end{aligned}$$

where $x_i'$ is the results of the classical update Eq. (2) and ${{\,\mathrm{sgn}\,}}$ is the usual sign function, i.e. 1 for positive argument and $-1$ for negative argument. The central parameter is $\eta$, which governs how expensive a change of opinion is. Additionally, we have to update the available resources of each agent in each time step by subtracting an amount proportional to the magnitude of the opinion change from its resources

4 $$\begin{aligned} c_i(t+1) = c_i(t) - \eta \left| x_i(t) - x_i(t+1)\right| . \end{aligned}$$

Note that using this definition $\eta$ can be compensated with a factor in front of the initial resources $c_i(0)$. Thus, we choose here $c_i(0)$ always such that its mean is $\left\langle c_i(0) \right\rangle = 0.5$ and treat $\eta$ as the free parameter without loss of generality. This model reduces to the standard heterogeneous HK model for $\eta = 0$.

Under these rules, an agent who has run out of resources is not able to move in the opinion space anymore and it therefore keeps its opinion for the rest of the simulation. However, it is still able to influence other agents. We will call it *a frozen agent* in the following.

These dynamical rules lead to a stable *final* state where the agents’ opinions stop evolving. We require as convergence criterion that the sum of all the changes is low enough. Specifically here this condition reads:

5 $$\begin{aligned} \sum _i \left| x_i(t+1) - x_i(t)\right| < 10^{-4}. \end{aligned}$$

We performed the simulations by the means of the algorithm introduced in our previous work^15^, which uses a tree data structure to speed up the simulation, while preserving correctness within the precision of the data type used to represent the opinion of each agent. This allowed us to carry out extensive simulations of systems sizes up to $n=32,768$ agents and still be able to explore the extended phase space given by $(\varepsilon _l, \varepsilon _u)$ and the cost $\eta$. Our results are issued from a statistical study over different realizations of the random initial conditions $x_i(0)$ and quenched disorder, $\varepsilon _i$, with 1000 to 10000 samples for each point of the parameter space $(\varepsilon _l, \varepsilon _u, \eta )$. However, the introduction of cost induces much longer convergence times than for the $\eta = 0$ case, therefore the very large sizes shown for the heterogeneous HK with no cost^15^, are still beyond reach.

### Clustering

The main question for the classical HK model is whether the society converges to consensus, splits into polarization with two opinions or fragments into many more opinions. Due to the averaging dynamics and the lack of noise in the classical model, the agents will group into very sharp clusters of practically the same opinion value, within the precision of the used data type (here single precision IEEE 754 floats and a tolerance of $10^{-4}$ to generously account for numerical errors), which are therefore quite easy to classify. So a good observable of the level of consensus is the mean size of the largest cluster.

**Figure 1:**
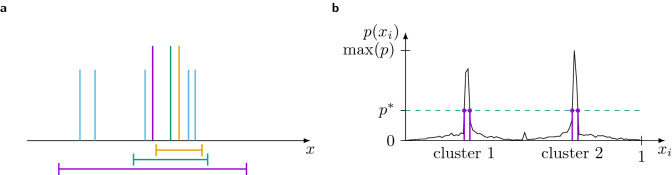
**(a)** Sketch to visualize the broadening of the clusters. Due to the frozen agents (light blue), agents with different confidences (horizontal bars below the opinion axis) interact with different sets of agents leading to different final positions. Note that each of the non-blue lines represents possibly multiple agents with similar confidence $\varepsilon _i$. **(b)** Histogram (with 100 bins) of the opinions of agents in the final state for a realization of $n=4096$ agents with a cost of $\eta = 4$. The dashed horizontal line is drawn at 1/3 of the maximum height. We classify two clusters from the four intersections of the dashed line with the peaks. All agents with a final opinion in the range marked by vertical violet lines are assigned to one cluster.

In contrast to those sharp clusters, the introduction of cost will result in broader, quasi-continuous distributions of opinions. The fundamental reason for this qualitative change lies in the presence of the frozen agents combined with the heterogeneity in the confidences. Similar to an effect already observed^15^, agents of different confidence $\varepsilon _i$ interact with different sets of frozen agents, which results in slightly different final states. This mechanism is sketched in Fig. 1a. In the limit of $n\rightarrow \infty$, this should actually result in a continuous spectrum of final opinions. The higher the fraction of frozen agents, the broader the peaks can become. As an example, for the broader peaks, a histogram giving the distribution of final opinions is shown in Fig. 1b.

Unfortunately, established methods to classify the clusters of a HK model, e.g. binning of the opinion space, do not work reliably with the relatively broad distributions. Therefore, this requires a new robust criterion, to define clusters of agents carrying the “same” opinion. The observable we use is loosely inspired by the *full width at half maximum* measure applied in, e.g. spectroscopy. From each final realization we create a histogram to estimate the probability density function *p*(*x*) of an agent to have opinion *x* in the final state, as shown in Fig. 1b. Then we classify the clusters using a threshold of $p^* = \frac{1}{3} \max p(x)$. The clusters are defined using the maximal intervals $[x_l, x_u]$, such that

6 $$\begin{aligned} p(x^\prime ) \ge p^* \;\forall x^\prime \in [x_l, x_u]. \end{aligned}$$

For each interval, the set of agents $\{ i \, | \, x_l \le x_i \le x_u \}$ constitutes a cluster. In other words, each peak larger than the threshold is identified as a cluster. The choice of the factor 1/3 to calculate $p^*$ is arbitrary but empirically chosen to be as small as possible while always being larger than the noise floor. Note that this method does not assign each agent to a cluster but it is suited as a robust estimate for the size of the largest cluster.

### Finite-size scaling

In the “Results” section, we will show that we find evidence of the existence of a second order phase transitions when the cost increases. In order to characterize it, we use well known finite-size scaling methods^21^, p. 232. For second order phase transitions, one expects the order parameter *S* to obey a *scaling* of the form

7 $$\begin{aligned} S(\eta , n) = n^{-b} f\left[ (\eta -\eta _c) n^a\right] , \end{aligned}$$

where *n* is the system size, and $\eta$, the cost of each opinion change, plays the role of a disordering field, an analogue to the temperature of thermodynamic systems. $\eta _c$ is the critical value and *a* and *b* are called critical exponents.

This means that the value of the order parameter changes in a region around $\eta _c$ from the typical value characterizing one phase to the typical value characterizing the other. This change becomes sharper but stays continuous for larger system sizes, i.e. the region where the order parameter changes becomes smaller. The scaling form Eq. (7) assumes that the region becomes smaller as a power law of the system size, such that a value of $a < 1$ means that the transition will be sharp in the $n \rightarrow \infty$ limit.

$f(\cdot )$ is the *scaling function*, and its most important property for our application is that it has no explicit dependency on the system size *n*. Thus, if we re-scale data measured for different system sizes *n* with the correct values of *a*, *b* and $\eta _c$ and plot it as $n^b S(\eta , n)$ over $(\eta -\eta _c) n^a$, all data points will collapse on the same curve *f*, in the critical region.

From a practical point of view, we estimate *a*, *b* and $\eta _c$, by an optimization of the curve collapsing. We use an established optimization method^22^, which has been successfully applied to critical phenomena^23–26^, and also to study scaling functions in other contexts^27^. The idea is to search in the $(a, b, \eta _c)$ parameter space for values such that a quality parameter *Q* is minimized. This quality parameter measures the average distance on the $(n^b S(\eta , n))$-axis of each data point from an estimate of the scaling form normalized by its standard error. The estimate is obtained by a linear fit through the data points of other sizes in that region. Conceptually this minimizes therefore the deviation of the data points from each other similar to fitting procedures which minimize a $\chi ^2$ value. Hence, this quality parameter per degree of of freedom has a very similar interpretation to a reduced $\chi ^2$ value: A quality close to 1 means that all data points lie on average one standard error off the estimated curve, which would be the best case one could expect. $Q \ll 1$ hints that the standard errors of the data are overestimated and $Q \gg 1$ means that the data does not fit well to the scaling assumption. Since we deal with finite system sizes and and data points are not taken exactly at $\eta _c$, values moderately larger than one are expected. Additionally this quality parameter *Q* can be used to estimate an error for the values of *a*, *b* and $\eta _c$, by finding the minimal and maximal values for *a*, *b* and $\eta _c$ at which the quality is $Q+1$, i.e. where the deviation is one standard error larger. We use an implementation of this optimization procedure from Ref.^28^.

## Results

**Figure 2:**
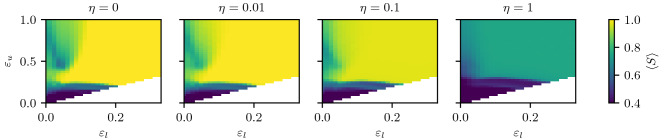
Phase diagrams of the consensus for different values of the cost $\eta$ in the $(\varepsilon _l, \varepsilon _u)$ plane determining the heterogeneity. Each image shows 1081 points $(\varepsilon _l, \varepsilon _u)$. Each point results from an average over 1000 samples of societies of $n=4096$ agents. Note the compressed colour range starting at $\left\langle S \right\rangle = 0.4$.

As an overview of the influence of the cost parameter, $\eta$, on societies having agents with confidences in different intervals, we show in Fig. 2$(\varepsilon _l, \varepsilon _u)$ planes of the phase diagram, for different $\eta$ values. The state of the system is measured by the means of the average size of the largest cluster $\left\langle S \right\rangle$, according to the clustering criterion introduced before.

The leftmost panel of Fig. 2 shows the previously studied^15^ case of $\eta = 0$ to serve as a baseline to observe the influence of cost. The most striking structure, is the *re-entrant consensus* region, around (0.05, 0.3). Also comparing this case with the results from using a strict clustering^15^ shows that this novel clustering is able to detect the same structures, validating the novel method.

For the very small value of the cost $\eta = 0.01$ the general shape remains, but the polarization region (green) at large $\varepsilon _u$ and low $\varepsilon _l$ becomes less sharp and the structures observed in the $\eta =0$ case, start to smear out. Also note that at small values of $\varepsilon _l$ but large values of $\varepsilon _u$, consensus actually is increased. We will explain this effect later in “Case C: Cost sightly improves consensus in fragmented systems”.

For small to intermediate costs $\eta = 0.1$ the re-entrant consensus is destroyed, but consensus is still reached when agents with large confidences, large values of $\varepsilon _u$, are included.

Finally, for large cost $\eta = 1$ there is no consensus at all, even above the critical value of $\varepsilon _l > 0.2$, where all the agents have a confidence larger than the critical value of the homogeneous HK model.

These observations naturally lead to the following questions: Is there a critical value $\eta _c$ above which consensus is suddenly lost? Or do we expect to lose consensus smoothly as $\eta _c$ increases? And in case where this threshold exists, what is the state for $\eta \ge \eta _c$? Is the society polarized, i.e. split in two clusters, or is there still a majoritarian cluster with a homogeneous floor of frozen agents?

**Figure 3:**
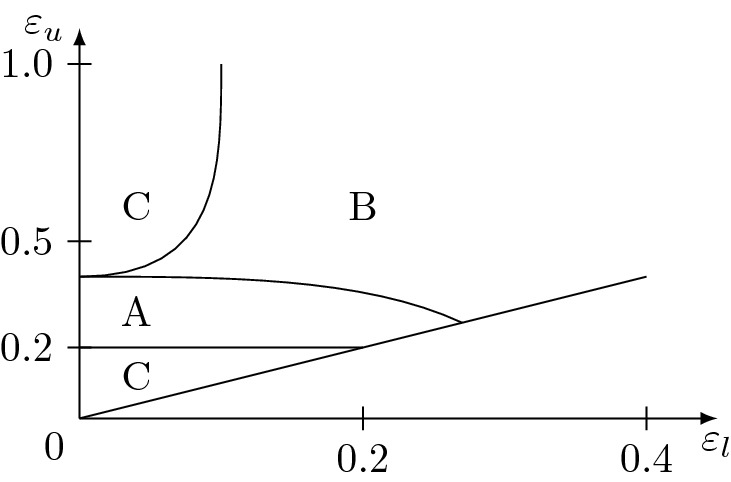
Regions of the $(\varepsilon _l, \varepsilon _u)$ space showing different behaviour with increasing $\eta$, issued from a dozen measurements, i.e. the lines are only a rough estimate.

Given the rich structure of the phase diagram of the heterogeneous HK model, we can split it into three main regions which show a qualitative different behaviour. For computational reasons, the precise determination of the borders of these regions is out of the scope of this work, we just give here a rough scheme of the identified regions in Fig. 3. The following results are issued from simulations done at a dozen points $(\varepsilon _l, \varepsilon _u)$ situated in these different regions for several values of $\eta$ and multiple system sizes.

In these three regions, we observe three qualitatively different classes of behaviour:

A. The most interesting case, which seems to greatly overlap with the region of the re-entrant consensus phase found in the case of zero cost^15^. There is strong consensus at $\eta = 0$ and increasing $\eta$ leads to an abrupt loss of consensus towards a polarization. Our analysis in “Case A: Transition to polarization with increasing cost”, shows that this is a phase transition of second order with a critical exponent, which seems to be the same over large parts of this region. Note that this behaviour is even present for homogeneous cases, e.g. $\varepsilon = 0.25$.
B. Increasing $\eta$ leads to a gradual loss of consensus. The final configuration consists typically of one single central majoritarian opinion and many agents frozen almost uniformly across the opinion space. The form of the curve $\left\langle S \right\rangle (\eta )$, i.e. the mean size of the largest cluster as a function of the cost, shows only very weak size *n* dependency (corresponding to a critical exponent of or close to 0, which means that this is not a sharp phase transition). The formation of a second cluster for a polarized configuration like in A, is suppressed since agents of one cluster could always see the other cluster and would travel towards the central opinion or freeze at different points on the way, according to their resources. The details of this region are presented in “Case B: Progressive loss of consensus with increasing cost”.
C. There is no consensus at $\eta = 0$ (see Fig. 2). With increasing $\eta$, frozen agents appear, but the opinion stays fragmented and no sharp transition can be observed. We will show some interesting peculiarities in “Case C: Cost sightly improves consensus in fragmented systems”.

### Case A: Transition to polarization with increasing cost

**Figure 4:**
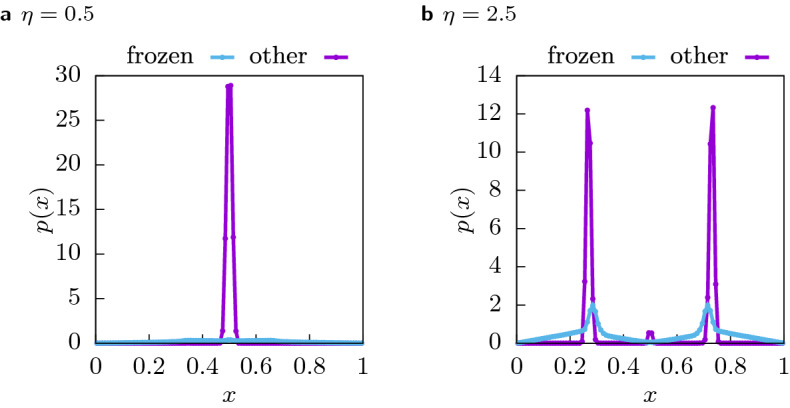
Histogram of the final opinion of agents with resources (violet) and without resources in the final state (light blue). The histogram was collected over 10, 000 samples of systems with $n = 16,384$ at $(\varepsilon _l, \varepsilon _u) = (0.1, 0.3)$ agents with **(a)** low costs of $\eta = 0.5$ and **(b)** high costs of $\eta = 2.5$.

First, we will analyse the distribution of final opinions to understand the role of the frozen agents in the equilibrium configurations of both phases. Figure 4 shows the distribution of final configurations for $(\varepsilon _l, \varepsilon _u) = (0.1, 0.3)$ and $n = 16384$ averaged over 10, 000 samples below and above the critical cost $\eta _c = 0.909(6)$ (see below). Frozen agents which have spent all their resources (light blue) and those which still interact as usual (violet), are shown. Figure 4a below the critical $\eta _c$ shows a clear consensus configuration with a single peak and almost no frozen agents. Figure 4b far above $\eta _c$, shows a clear two-peak structure, indicating polarization. Indeed, even for this extremely high cost, most agents still have resources in the final state, and even a large fraction of the frozen agents, i.e. those without resources, are located within the bounds of one of the two clusters. The small peak in the centre (also visible in the single realization shown in Fig. 1) is caused by agents ending up with a central opinion because, given their confidence values, they can interact with both peaks and end up with the average opinion of both clusters.

#### The dynamics

**Figure 5:**
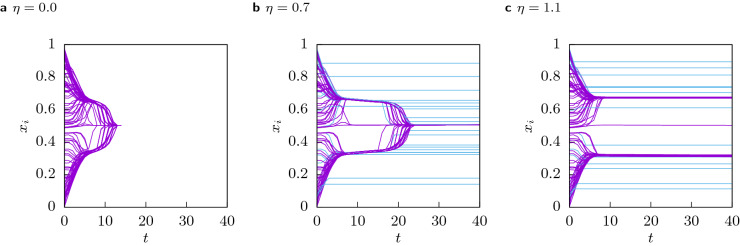
Single trajectories of the same system with $n = 16384$ agents for different values of the cost: **(a)** $\eta = 0$, **(b)** $\eta = 0.7$, **(c)** $\eta = 1.1$. To improve the visualization only the trajectories of 100 randomly sampled agents are shown in the figure.

In Fig. 5 three examples of trajectories of the same system at $(\varepsilon _l, \varepsilon _u) = (0.1, 0.3)$ with the same initial opinions $x_i(0)$, the same confidences $\varepsilon _i$ and the same initial resources $c_i(0)$, but with different values of the cost $\eta$ are shown. Note that the system has $n = 16384$ agents but for clarity of the figures only the trajectories of 100 randomly chosen agents are visualized. Figure 5a shows the dynamics of the system at $\eta = 0$. We can observe the ‘bell’ structure^15^ which facilitates consensus despite the high proportion of very closed minded agents. This system converges to total consensus after $t=15$ steps. Figure 5b shows the influence of a low cost: Multiple agents freeze outside of the ‘bell’, such that the two branches are pulled away from each other leading to an increase in the time needed to reach consensus. Note that this effect is caused by agents with very low initial resources $c_i(0)$ and we will see in “Influence of the distribution of resources” that the exclusion of of agents with initial resources close to zero drastically changes the character of the transition. With increasing cost more agents freeze outside of the ‘bell’ such that the branches are pulled stronger away from the centre, preventing consensus to form, as show in Fig. 5c.

#### The order parameter

We study the behaviour of the mean relative size of the largest cluster $\left\langle S \right\rangle$ as a function of $\eta$. In the consensus case, at $\eta = 0$, one expects $\left\langle S \right\rangle = 1$. In the polarization phase, one expects $\left\langle S \right\rangle \le 0.5$, since a few frozen agents will be scattered outside of both clusters.

**Figure 6:**
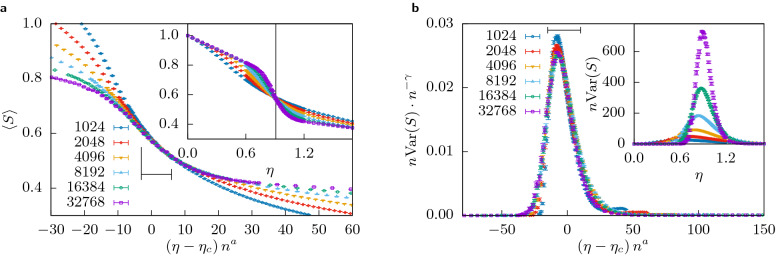
**(a)** Inset: The mean size of the largest cluster $\left\langle S \right\rangle$ (cf. “Clustering”) for multiple system sizes $1024 \le n \le 32768$ as a function of the cost $\eta$ for $(\varepsilon _l, \varepsilon _u) = (0.1, 0.3)$. Each data point is averaged over 10000 samples. Note that the density of data points is higher close to the transition point and that error bars are smaller than the symbols. Main Plot: The same data but with rescaled axes (cf. “Finite-size scaling”). The black marker shows the range over which the quality of the collapse *Q* was optimized. The exponent for the vertical axis was fixed at $b=0$, the value for the critical point and the critical exponent *a* as obtained by the optimization are listed in Table 1. **(b)** The same but for a quantity analogue to the susceptibility $\chi = n {{\,\mathrm{Var}\,}}(S)$.

The inset of Fig. 6a shows $\left\langle S \right\rangle (\eta )$ for a selection of system sizes *n*. The curves become steeper for larger system sizes. This behaviour is typical of second order phase transitions. Since our order parameter, $\left\langle S \right\rangle$, is dimensionless, it is expected that they cross at the critical value of the control parameter $\eta _c$ (cf. e.g. percolation or cumulants for magnetic transitions), which is indeed the case here. Also this means that we do not have to scale the vertical axis, hence $b=0$.

#### Scaling behaviour

In order to find the critical parameter $\eta _c$ and critical exponent *a*, we apply the optimization technique described in “Finite-size scaling” such that curves for all system sizes collapse on the common scaling function when the axes are appropriately rescaled. This is shown in the main plot of Fig. 6a. Using the values $a = 0.45(4)$ and $\eta _c = 0.909(6)$, it is possible to collapse all curves on the common scaling function. In the range marked by black lines on the figure, the curves for $n > 4096$ deviate on average by 0.39 standard errors from each other. Considering the very small statistical errors in the data (the error bars are always smaller than the symbols), this indicates a very good quality of the collapse.

The concept of universality in the context of phase transitions means that the fundamental descriptors of the transition, i.e. the critical exponents, are robust against details of the model and only depend on the symmetries of the interactions or the dimension of the system. Here, we see hints of universality in regards to the values of $(\varepsilon _l, \varepsilon _u)$, as long as we are still in region A of the phase diagram.

The inset of Fig. 6b shows the behaviour of the fluctuation of the order parameter $\chi = n {{\,\mathrm{Var}\,}}(S)$ (the analogue to the susceptibility of magnetic systems), which exhibits all characteristics expected at criticality: it diverges with system size, its peak moves towards the critical point for increasing system sizes and it can be collapsed using a second critical exponent $\gamma$ to scale the vertical axis.

Note that the collapse in the main part of Fig. 6b is obtained by an independent optimization over the region $[-15, 10]$ of the rescaled horizontal axis, for sizes $n \ge 8192$, yielding $\eta _c = 0.970(6)$, $a = 0.45(1)$ and $\gamma = 0.987(5)$ with a quality of $Q = 5.2$. As usual, the collapse of a response function is not as good as for the order parameter $\left\langle S \right\rangle$. In fact, the quite large quality factor hints that the sizes might not be large enough to get a reliable estimate which probably causes the estimated critical value to deviate beyond error bars from the one obtained by the collapse of the order parameter. Therefore the value obtained from the collapse of the order parameter should be considered as a better estimate for the actual critical value. Nevertheless, the optimized collapse still yields *a* and $\eta _c$ which deviate less than $10\%$ from the values given in Table 1. As the deviations are mainly observed for the smallest sizes and a convergence to the common curve is visible as size increases, it is expected that the results will meet those obtained for $\left\langle S \right\rangle$ in the thermodynamic limit.

**Figure 7:**
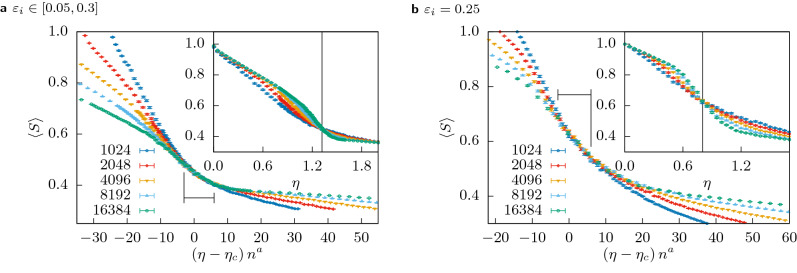
Insets: The mean size of the largest cluster $\left\langle S \right\rangle$ for multiple system sizes $1024 \le n \le 16384$ as a function of the cost $\eta$ for **(a)** $(\varepsilon _l, \varepsilon _u) = (0.05, 0.3)$ and **(b)** $\varepsilon _i = 0.25$. Each data point is averaged over 1000 samples. Main plots: The same data but with rescaled axes. The black marker shows the range over which the quality of the collapse *Q* was optimized. The exponent for the vertical axis was fixed at $b=0$, the value for the critical point and the critical exponent *a* as obtained by the optimization are listed in Table 1.

Figure 7 shows the same rescaling for different points of region A including the homogeneous case, shown here for $\varepsilon _i = 0.25$. The critical point and exponent were determined using the same technique as before and all values are compatible within statistical errors. Their exact values as well as used range and the quality are shown in Table 1. In “Influence of the distribution of resources” we will explore the robustness of this phase transition against changes of the distribution of initial resources, $c_i(0)$, and we will show a particular case in which this universal behaviour may be destroyed.

**Table 1:** Critical values $\eta _c$ and exponents *a* obtained with the optimization approach from “Finite-size scaling” (with a quality of *Q* over the *range* on the rescaled axis) for different values of $(\varepsilon _l, \varepsilon _u)$ and different distributions of the initial resources $c_i(0)$. The critical exponents *a* are robust to changes in the initial resource distribution as long as there are still agents with very little starting resources. Note that always the mean initial resources $\left\langle c_i(0) \right\rangle = 0.5$.

	$(\varepsilon _l, \varepsilon _u)$	$\eta _c$	*a*	*Q*	Range
$c_i(0) \in U[0, 1]$	(0.10, 0.30)	0.909(6)	0.45(4)	0.39	$[-3, 6]$
$c_i(0) \in U[0, 1]$	(0.05, 0.30)	1.32(1)	0.42(5)	0.89	$[-3, 6]$
$c_i(0) \in U[0, 1]$	(0.25, 0.25)	0.80(2)	0.41(6)	1.05	$[-3, 6]$
half-Gaussian	(0.10, 0.30)	0.73(1)	0.43(2)	0.76	$[-3, 6]$
$c_i(0) = 1 - (\varepsilon _i - \varepsilon _l) / (\varepsilon _u - \varepsilon _l)$	(0.10, 0.30)	0.63(1)	0.43(1)	1.06	$[-3, 6]$

#### Influence of the distribution of resources

The results discussed so far were obtained for a uniform distribution of initial resources among the population. This simplifying hypothesis being quite unrealistic, we study here whether the obtained results are robust face to different distributions of initial resources.

Let us consider first, that initial resources $c_i(0)$ are distributed according to a half-Gaussian

8 $$\begin{aligned} p(c) = \frac{\sqrt{2}}{\sigma \sqrt{\pi }} \exp \left( - \frac{c^2}{2\sigma ^2} \right) , \; c>0. \end{aligned}$$

**Figure 8:**
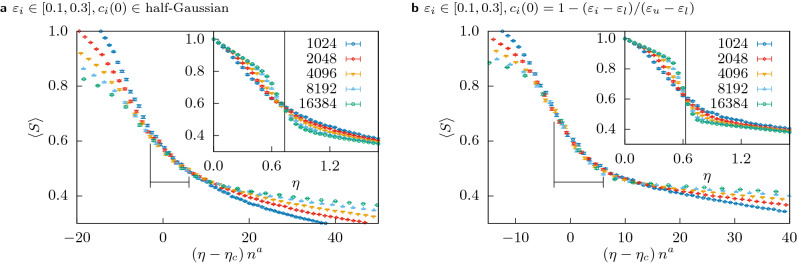
Insets: The mean size of the largest cluster $\left\langle S \right\rangle$ for multiple system sizes $1024 \le n \le 16384$ as a function of the cost $\eta$ for $(\varepsilon _l, \varepsilon _u) = (0.1, 0.3)$ with initial costs $c_i(0)$**(a)** drawn from a half-Gaussian distribution and **(b)** deterministically chosen as a function of their confidences $c_i(0) = 1 - (\varepsilon _i - \varepsilon _l) / (\varepsilon _u - \varepsilon _l)$. Each data point is averaged over 1000 samples. Main plots: The same data but with rescaled axes. The black marker shows the range over which the quality of the collapse *Q* was optimized. The exponent for the vertical axis was fixed at $b=0$, the value for the critical point and the critical exponent *a* as obtained by the optimization are listed in Table 1.

In order to keep the system comparable with the previous case, we chose $\sigma = \frac{\pi }{2\sqrt{2}}$, such that the mean resources per agent $\left\langle c_i(0) \right\rangle = 0.5$, like before. So, here agents with very little resources are more probable and the richest agents might have $c_i(0) > 1$. Still the qualitative behaviour is the same as before: we observe in the inset of Fig. 8a a phase transition at a slightly higher critical value. Also the optimization based collapse method yields the same universal exponents within error bars, which are shown in Table 1.

In the previous cases the resources and the confidences were uncorrelated. In order to explore the situation where they are correlated we assume the simplest hypothesis:

9 $$\begin{aligned} c_i(0) = 1 - \alpha (\varepsilon _i - \beta ), \end{aligned}$$

where again, in order to keep the system comparable to the previous cases, $\alpha$ and $\beta$ are chosen such that $c_{\mathrm{min}} = 0$ and $\left\langle c_i \right\rangle = 0.5$. This leads to $\beta =\varepsilon _l$, $\alpha = 1 / (\varepsilon _u - \varepsilon _l)$. (Note that a similar proportional connection $c_i(0) = \alpha (\varepsilon _i - \beta )$ lead to almost identical results as the uniform initial resources.) The results show no qualitative deviation from the uncorrelated case, the phase transition is shown in the inset of Fig. 8b and the critical exponents are compatible with those of Table 1. The critical exponents are therefore robust face to correlations between confidences and resources.

Finally, a different qualitative situation occurs for a society without very poor agents (those with $c_i(0) \simeq 0$). Note that this is automatically the case for power-law distributions, which generally have a lower cutoff.

**Figure 9:**
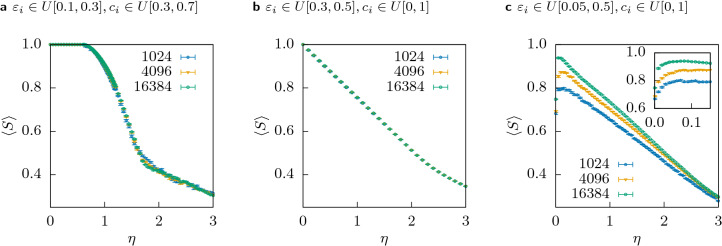
Behaviour of the size of the largest cluster with increasing cost for **(a)** Region A, but with a distribution of the cost, which does not include very poor agents, i.e. $c_i$ drawn uniformly from [0.3, 0.7]. **(b)** Region B. The decrease of the largest cluster size is independent of the system size, such that we observe even in the infinite system a gradual decline instead of a a sharp transition. Note that for the above cases the data points for different system sizes lie almost on top of each other, such that only the largest size might be visible. **(c)** Region C. At $\eta = 0$ there is no consensus, i.e. $\left\langle S \right\rangle (0) < 1$, the larger the system, the smaller the largest cluster. The inset shows a zoom on small values of $\eta$ visualizing that in some fringe cases a small cost can increase the level of consensus in a society.

Figure 9a shows the results for a system where the resources are uniformly distributed but within boundaries that prevent the existence of very poor agents, here $c_i(0) \in U[0.3, 0.7]$. Note that here the upper cutoff is shifted to 0.7, again in order to keep the same mean initial resources $\left\langle c_i(0) \right\rangle = 0.5$ like before. The confidences are in the region where a consensus re-entrant phase is observed for a system without cost. Interestingly, we observe a decrease of $\left\langle S \right\rangle$ which seems independent of the size *n*, suggesting an exponent close to zero, therefore the transition never becomes sharp. Note that the lack of very poor agents leads to different dynamics. Since all agents have enough resources to travel $c_{\mathrm{min}}/ \eta$ in opinion space, for moderate values of $\eta$ not a single agent will freeze outside of the ‘bells’ that are observed in the trajectories of a single realization, in contrast to the case we studied before in Fig. 5. Agents frozen inside of the bell pull the two branches towards each other, which helps consensus. This happens for all the system sizes. When $\eta$ is finally large enough to freeze agents outside of the branches, a very large fraction of agents is frozen (for $c_i(0) \in U[0.3, 0.7]$ almost $50\%$) which leads to a rapid change to polarization, again without relevant size dependence.

### Case B: Progressive loss of consensus with increasing cost

Region B of Fig. 3 corresponds to the region of the phase space, where consensus is achieved extremely quickly in the $\eta =0$ case. Introducing a cost does not lead to polarization as in region A, since the confidences, i.e. the interaction range of the agents, are too large allowing the two polarized clusters to interact and converge to consensus. So the final configurations in this case consist even for very high cost of a single large central cluster and many frozen agents, which are spread in the whole opinion space (their density increasing linearly towards the central cluster).

In fact, we can observe in Fig. 9b that the size of the largest cluster decreases linearly in $\eta$ independent of the system size. This behaviour is plausible considering that the number of agents with $c_i(0) < \eta \left| 0.5 - x_i(0)\right|$, which do not have enough resources to reach the central consensus opinion and freeze outside of the central cluster, increases linearly. Therefore, in region B the introduction of cost does not lead to a phase transition but to a linear decrease of the size of the largest cluster.

### Case C: Cost sightly improves consensus in fragmented systems

The last class of different behaviour we observed occurs in region C. The main effect we observe in the behaviour of $\left\langle S \right\rangle$ in Fig. 9 is a similar linear decrease with $\eta$ like in region B, but starting at lower values, since consensus is already absent at $\eta = 0$.

An interesting effect we observe is that the introduction of a small cost can actually increase the mean size of the largest cluster $\left\langle S \right\rangle$ with respect to the $\eta =0$ case, paradoxically, increasing consensus. Figure 2 shows this effect, where the top right corner of region C around (0.1, 1.0) becomes lighter, i.e. more consensus, from $\eta = 0$ to $\eta = 0.01$. And also the inset of Fig. 9c shows this phenomenon. This contra-intuitive behaviour is caused by frozen agents which freeze in between two clusters of the fragmented opinion space and act as a bridge: both clusters see the frozen agent and are attracted, such that at some point they can interact with each other and end up on the same opinion cluster.

## Conclusions

In this article we investigate the collective effects of the introduction of cost in an opinion dynamics model. The stylized model proposed here addresses the situations where changing our opinion is not straightforward but requires some effort instead. As a consequence, the possibility for an agent to change its own opinion is conditioned to the resources it has. In particular, in this model, after spending all its resources the agent cannot evolve anymore but it is still part of the society and its static position may influence the behaviour of others. Given form of Eq. (4) it could be also thought in terms of an amount of resources per unit time allocated to the agents, where the fixed parameter $\eta$ would stand for a fixed rate of expenses.

We have coupled the dynamics of the heterogeneous Hegselmann-Krause model with that of the resources of the agents, which decrease with time as the agent evolves in the opinion space. Intuitively, one expects that the introduction of cost would lead, as a trivial consequence, to the reduction of the size of the majoritarian opinion group, due to the agents that freeze in different regions of the opinion space. Instead, the results of the extensive simulations we have performed in the parameter space ($\varepsilon _l, \varepsilon _u, \eta$) show a much more complex behaviour depending on the region of the parameter space we are studying.

The most outstanding result is the way in which the outcomes of the dynamics of a society that reaches consensus in the standard HK model is modified. When the agents have large confidences, the introduction of cost gradually reduces the size of the largest opinion group, as expected. When, on the contrary, the society contains agents with intermediate confidences, a second order phase transition is observed. By a finite-size study, we have been able to characterize this transitions by the critical exponents describing the behaviour of the order parameter and its fluctuations and we have observed that they are universal, independent of the details of the resource distribution, with one interesting exception, which constitutes the second interesting result. If the initial distribution of resources guarantees that every agent in the society has a minimum amount of resources, then no phase transition is observed and the effect of increasing the cost simply leads again to a smooth decrease of the large opinion cluster.

The interpretation of these results is interesting because it means on one hand, that the structure of the population, in terms of the confidences of the agents, is relevant in order to evaluate the consequences of the introduction of the cost of opinion change: very confident societies will not experience a phase transition due to an increase of the cost of opinion change, while societies with intermediate confidences will undergo such sharp transition due to an increasing cost.

On the other hand, in the case of a society with intermediate confidences, an initial distribution of resources that does not include very poor agents guarantees that there is no sharp transition when the cost increases.

This study opens different prospective lines of research, like the role of a networked society or the inclusion of the possibility for the agent to choose whether to change its opinion according to the amount of its remaining resources. Moreover some modifications to this model could be made to examine some cases not considered here, like the case where the maintenance of an opinion needs to be payed from fixed resources, which could be valuable to study exhaustion effects, or the situation where a fixed cost is paid at each opinion change while allowing for the replenishment of the resources after some time. Finally it would also be interesting to study the coupling of the cost and the opinions, breaking the equivalence of the opinions of this model by making some of them more costly than others.
